# Colleagues’ Work Attitudes towards Employees with Disability

**DOI:** 10.3390/ejihpe13010009

**Published:** 2023-01-10

**Authors:** Sara Santilli, Maria Cristina Ginevra, Laura Nota

**Affiliations:** Department of Philosophy, Sociology, Education and Applied Psychology, University of Padova, 35100 Padova, Italy

**Keywords:** disability, employee attitudes, work inclusion, colleagues

## Abstract

Background: The present study investigates the significance of work inclusion in people with a disability and then aims to examine colleagues’ attitudes. Considering Stone and Colella’s model, we analyzed the colleagues’ attitudes and variables related to the disability, such as type of disability and type of presentation of colleagues with disability, and colleague’s characteristics, such as gender, educational level, and experience in work with people with disability. Method: We randomly assigned two hundred eighty-six employees to a standard condition (hypothetical colleagues with a disability presented by the impairments labels) or favorable condition (hypothetical colleagues with a disability presented by their past work experiences). Results: The type of disability and its presentation influence colleagues’ attitudes. Besides employees’ gender, educational level and experience in work with people with a disability influences the attitudes toward them. Conclusions: Implications for practice were discussed.

## 1. Introduction

In recent years, many contributions describe the benefits of inclusive social, work and school contexts that recognize heterogeneity, equity, and co-operation [1]. These contests are crucial because they can affect the quality of life positively and promote the co-construction of strength and the active participation of all. Given the current socioeconomic challenges facing our society, in addition to globalization and multi-diversity, the need to co-construct inclusive contextx based on mutual support, solidarity, and to build relationships and protective networks is invoked as an essential determinant of the well-being of individuals [2,3,4].

Therefore, inclusion concerns the context and the people who are occupying it; the right to participate in an inclusive physical, social and psychological place, able to guarantee to every person, with its uniqueness, the active participation in social and civic life [4]. In these contexts, policies are promoted that seek to value heterogeneity and satisfy the unique needs of people, with particular attention to people with vulnerabilities. Moreover, Wasserman and colleagues [5] highlight that an inclusive society exists when all people have the chance to be present and to participate in core activities on behalf of the collective. Positive public attitudes toward people with a vulnerability can impact the abilities of all to form new social relations with other individuals in the population, developing a series of mutual influences [6]. Different studies emphasize more inclusive programs that are associated with more positive attitudes toward people with a vulnerability [1].

Specifically related to work context, Shore and colleagues [7] define work inclusion as the degree to which workers distinguish themselves as esteemed members of the workgroup through feeling satisfied in belongingness and uniqueness. Similarly, Lirio and colleagues [8] (p. 443) referred to inclusion as “when individuals feel a sense of belonging, and inclusive behaviors such as eliciting and valuing contributions from all employees are part of the daily life in the organization”. Furthermore, Avery and colleagues [9] stated that inclusion is the extent to which employees believe their organizations engage in efforts to involve all employees in the mission and operation of the organization concerning their abilities.

The benefits of work inclusion at different levels, i.e., for individuals with a disability, for the workplace, and the overall social context have been highlighted in different studies [10,11,12]. 

As regards the benefits for individuals with a disability, work can be considered as a place of socialization and an instrument that supports the definition of one’s personal position in the community. Therefore, it contributes to the development of elevated levels of self-esteem, mental health and wellbeing [13,14], promoting positive career identity and life satisfaction [15,16].

With regard to the workplace, several studies in the diversity organization field undoubtedly highlight the advantages of inclusion and diversity in the work context, positively affecting team creativity and team engagement, and encouraging the search for original information and perspectives, leading to better decision-making and problem-solving [17,18,19,20].

Lastly, as regards society, the Organization for Economic Co-operation and Development (OECD) high-level policy forum entitled *Sickness, Disability, and Work* [21] emphasized that the work participation of people with disabilities impacts the overall economy in a positive manner. As Nunnerley and colleagues [22] underscored, increasing employment chances for working-aged people with disabilities may improve their wellbeing status and thus reduce health care costs. 

With the significant benefits of work inclusion of people with disabilities and vulnerabilities, it is necessary to analyze the factors that inhibit inclusion in the work context. 

Even though a job is an essential condition for decreasing social exclusion, even people with disabilities who are employed frequently indicate that they feel stigmatized and less included in their colleagues’ groups than workers without disabilities [13]. Therefore, Vornholt and colleagues [13] proposed that merely getting a job is not sufficient, and that colleagues’ positive attitudes are an essential pre-condition for work inclusion [14,20]. 

Other researchers have found that indifferent or unfriendly attitudes toward working with employees with disabilities held by coworkers without disabilities could also have significant effects that may lead this population to fail or become unable to maintain their jobs or to experience a lower quality of life [23]. Ferguson and colleagues [24] and Hsu and colleagues [25] also found that employees without disabilities initiated more interactions, such as teasing and joking among themselves, than they did with their coworkers with an intellectual disability.

Stone and Colella built a model of variables influencing the attitudes towards individuals with disabilities in organizations [26,27]. This broad model covers factors including the environmental, organizational, and personal. According to the model, organizational characteristics and legislation influence the attributes (e.g., demographic attributes, personality, gender, and type of disability) of the employees with and without disabilities as well as the nature of the jobs (e.g., skill requirements, interrelationship, reward system). These variables, in turn, relate to psychological outcomes, such as labeling or emotional states, and job-related anticipations of colleagues concerning an employee with a disability. Expectations and outcomes then affect the behavior of the employee with a disability and produce responses toward this treatment.

The current study builds on this model and contributes to it by measuring colleagues’ attitudes and investigating the type of disability; the type of presentation (a focus on disability versus a focus on previous work experience); colleagues’ characteristics; and previous experience in working with people with a disability.

Type of disability. Aspects associated to workers with disabilities are prognostic of inclusion effects of a member with a disability [12,26,27]. Some research papers observed an influence of the nature of the disability on attitudes toward work inclusion of workers with a disability. For example, McLaughlin and colleagues [28] revealed that a disability has a non-direct influence on attitude. They specifically found that with increasing severity and controllability of the disability, negative attitudes toward people with a disability increase. Lau and Cheung [29] state that people with mental health issues are subjected to greater levels of prejudice than those with an intellectual disability. Fevre and colleagues [30] observed that having an intellectual or emotional disability increased the likelihood of detrimental treatment in the workplace by 117% compared to 15% for physical impairments. Nota and colleagues [4] found more negative employer attitudes towards people with a psychological problem than candidates with intellectual and sensory disabilities, highlighting that the manner of psychological difficulties denote the situation most connected with an unfavorable opinion.

Type of presentation. Colella and colleagues [31,32], Ren and colleagues [33], and Nota and colleagues [4], observed that when people with a disability were described via narratives of their previous job activities, attitudes on performance were more positive than when people with a disability were described via narratives that did not involve their previous job information.

Colleagues’ characteristic. Some findings present a significant effect of gender on attitudes towards people with a disability [34,35,36,37], while other studies find no effect at all [4]. Jones et al. [35] report that men were more discriminatory toward employees with disabilities than were women.

For the factors of age and education, no definite conclusion can be drawn from the results of the research conducted to this point.

Type of previous experience. Different studies highlight that individuals with past knowledge about disabilities held more favorable attitudes than did the general population [38,39,40]. McManus and colleagues [41] and Nota and colleagues [4] argue that the quality of interaction is another crucial component to consider in attitude research.

Despite the importance of colleagues in the construction of inclusive work environments, from a study conducted by Wilson and Scior [42], there seems to be a low focus on colleagues. Considering 540 articles generated from the electronic database related to attitudes toward people with disabilities, over half of those participants were undergraduate and postgraduate students, and attitudes were analyzed in only 85 employees who worked with (or were in close relation with) individuals with disabilities. Considering the role that colleagues have in making their colleagues with disabilities feel included, we decided to design a study to analyze their attitudes toward co-workers involving employees with and without the experience of an inclusive work context.

Consequently, were also hypothesized that, related to the impairments, the colleagues would show more positive attitudes towards people with a smaller amount of severe disability and in particular towards individuals with sensory disabilities (SD) and, in particular: (a) as regards ‘type of disability’, a more positive attitude for both job performance and social acceptability, and more negative attitudes towards the applicant presenting psychological problems (PP), at least in terms of social acceptability; (b) Regard ‘type of presentation’, a more positive attitude in the presence of descriptions highlighting the applicants’ previous work experience. Concerning colleagues, it was assumed that they would show (c) a more positive attitude on behalf of female colleagues; (d) a more positive attitude by colleagues with higher educational levels; and (e) a more positive attitude by colleagues with previous experiences of people with disabilities in the work context.

## 2. Materials and Methods

### 2.1. Participants

We recruited 286 Italian employees working in mid-size Italian industry metal mechanical and agri-food businesses (50–250 employees and annual sales less than EUR 50 million): 126 men (44%) and 169 women (60%) aged 19–60 years (mean age = 39.25; SD = 10.66). A total of 165 (58%) were employed as white collar workers, and 121 (42%) were blue collar workers. With regard to previous contact and experience with people with disabilities, 100 (35%) workers reported having experience with colleagues with a disability, and 186 (65%) reported that they had not. Concerning educational levels, 19 employers (6%) had received a middle school diploma; 29 (10%) had obtained a three-year high school diploma, 158 (56%) had obtained a three-year high school diploma, and 80 (28%) had a university degree.

### 2.2. Instrument

‘Work for people with disability’ is a questionnaire which examines work colleagues’ attitudes towards people with a disability. The instrument was elaborated based on the measure by Nota and colleagues [4], and focuses on employers’ attitudes. The questionnaire, in line with Nota et al.’ s measure [4], presented descriptions of three hypothetical work colleagues with a disability: the first was characterized by an SD; the second by an intellectual disability; and the third with PP. Ten 7-point scale items followed which were designed to examine the work colleagues’ attitudes towards workers with a disability. 

Santilli and colleagues [11], using using the questionnaire in a pilot study, showed a two-factor solution. The first factor (31.17% of the variance), Work Performance, was composed of six items and concerned attitudes about work performance (Cronbach’s alpha = 0.80). The second factor (13.21% of the variance), Social Acceptability, comprised three items and reflected attitudes towards the candidate’s potential for being socially accepted (Cronbach’s alpha = 0.72) and showed a moderate positive correlation with life satisfaction (0.43). In this study, the internal reliability was 0.79 and 0.71.

The two average scores of the Work Performance and Social Acceptability subtests were used to verify our hypotheses as attitude indicators for each disability (the higher the value, the more positive the attitude. 

### 2.3. Experimental Design

As suggested by Nota and colleagues [4] and by Biggs and colleagues [43], workers were randomly designated to one of two experimental conditions: one in which candidates with a disability were presented by mentioning the disability (standard presentation condition: focus on disability) and one in which the applicants were also presented with a reference to their positive aspects (positive presentation condition: focus on strengths). Among the workers, 149 (52%) were randomly assigned to the ‘standard presentation’ condition and 137 (48%) to the ‘positive presentation’ condition. As regards, the ‘standard presentation’ condition, for example SD, participants received the following description:

Serena is a young adult with a severe hearing disability; in addition to not hearing, she only emits some sounds, often difficult to understand. She has difficulty reading and understanding argumentative texts. She attended a professional institute and graduated from high school. She had the opportunity to do internships in the company. The work colleagues of the past described her as a person with difficulty in understanding the verbal expression, but they also say that she was calm and self-controlled.

Only the participants in the ‘positive presentation’ condition received, for example, the following additional information in the instructions: ‘They also said that she was a willing young woman, who did what was asked with clear written instructions’.

### 2.4. Procedure

The workers were identified by contacting local business associations for two provinces in northeastern Italy. In a preliminary step, the workers were contacted by phone and were informed as to the purpose of the study. Furthermore, it was clarified that the participation in the research was voluntary, and that the questionnaires could be filled in anonymously. The questionnaires were distributed through an e-mail message in which the link to the online questionnaire was provided. Approximately 98% of the workers completed the questionnaire and sent it back. 

Ethics Statement. No IRB format is required All procedures performed in studies involving human participants were in accordance with the ethical standards of the Italian Association of Career Guidance (SIO), and the Italian Association of Psychologists (AIP). Specifically, the ethical code of the Italian Association Psychology, which was approved in 2015 and revised in 2022, emphasizes that with regard to psychological research with human beings, the Ethics Committee pays special attention to researches involving (a) a risk to the psychological and physical well-being of participants; (b) the participation of vulnerable persons (such as minors, persons unable to express consent, imprisoned persons, hospitalized or institutionalized persons; groups exposed to stigma or risk of social discrimination); (c) the use of biomedical apparatus and invasive investigative tools; (d) the use of deception; (e) the use of stimuli that may hurt the personal and cultural sensitivities of the persons participating; and (f) the introduction of limitations on the right to anonymity and confidentiality of participants. This study does not fall into any of these categories; however, it has been developed and implemented respecting all rules of conduct under the code of ethics: information and consent for participation in research (article 1); return of results (article 3) through a personalized report for each participant; and respect of privacy and anonymity (article 4). 

Informed consent: Informed consent was obtained from all individual participants included in the study.

### 2.5. Data Analysis

To test our hypothesis, we followed the regression procedure used by Le Blanc and colleagues [21]. To do this, we created dummy variables representing disability (1 = SD; 2 = intellectual disability; 3 = PP), gender (0 = male; 1 = female), education level (1 = middle school diploma; 2 = high school diploma; 3 = bachelor’s degree; 4 = master’s degree), previous experience (0 = no experience; 1 = previous experience) and type of presentation (0 = neutral presentation; 1 = positive presentation).

## 3. Results

We first conducted a preliminary analysis of the data. Table 1 presents the intercorrelations among the measures. 

The means, standard deviations, and correlations for all study variables are reported in Table 1. Positive and significant correlations were observed among work performance and social acceptability attitudes and the type of presentation of disability. Work performance attitudes also correlate positively with educational level. As regards social acceptability attitudes, this also correlates positively with gender and the previous experiences of colleagues, and negatively with the type of disability. 

Table 2 presents the means and standard deviations for participants in the work performance and social relation with SD, intellectual disability, and PP. 

Next, we examined the statistical significance of change on the outcome variables of work performance and social acceptability. 

The results of the regression analysis for work performance attitudes revealed that they increase with the higher educational level of participants and with a positive presentation of colleagues with disabilities (see Table 3; Model 1). Specifically, there was a significant effect for educational level (β = 0.071, *p* = 0.043), and type of presentation (ß = 0.128, *p* = 0.01).

As regards social acceptability attitudes, the results of the regression analysis revealed that employees show more negative attitudes toward colleagues with PP than their colleagues with SD or intellectual disabilities (β= −0.42, *p* = 0.001). The results also highlight the more positive social acceptability attitudes in women (ß = 0.116, *p* = 0.01) than men, and in colleagues with higher educational levels than women (ß = 0.103, *p* = 0.01), and andin women with previous experience (ß = 0.071, *p* = 0.02) (see Table 3; Model 2).

## 4. Discussion

Work is essential in peoples’ lives, but for people with disabilities, employment might be hindered by the negative attitudes of their colleagues. Our premise is that if colleagues display inclusive behavior, people with disabilities should become active agents in co-constructing their work teams and environments. Therefore, this study was aimed to better understand which variables characterize the employees’ attitudes toward colleagues with a disability based on Stone & Colella’s model [27] and previous studies in this field [4,44].

As regards the first hypothesis concerning the type of disability, employee attitudes were more negative towards people with PP than colleagues with an intellectual disability and SD, especially in terms of social acceptability. This finding is in line with literature showing how psychological problems represent the condition most frequently associated with a negative view [45].These differences in the attitudes reported concerning the three groups of disability can be explained by the fact that attitudes differ between disease level and disabilities [46,47]. Beyond the above conceptual explanation, the differences in attitudes between the disability groups provide support for the disability, whereby people with mental illness are less socially desirable and people with intellectual disabilities and SD are accepted less. 

With regard to the type of presentation variable, extra information associated with candidates’ strengths and proper behavior in previous work experiences is related with more positive colleagues’ attitudes in work performance and social acceptability, as compared to the results in the condition where only the disability label was assumed. This result is related to the study conducted by Nota and colleagues [4], in which the main effect of the candidates’ type of presentation (‘standard’ versus ‘positive’) has resulted in a group of employers that showed more positive attitudes in the ‘positive presentation’ condition. According to some authors, referring to the strengths of people with disabilities starts a process of assigning a series of positive characteristics which, in turn, can promote social closeness [19,48]. Concerning the variable of employee’s characteristics, the women employees rated the social acceptability levels of workers with a disability more positively than they did in the work performance attitudes. This finding is in line with Vornholt et al. [13] They found that women, in general, experienced the least social distance from people with disabilities.

Furthermore, McDonnall and colleagues [49] and Nota and colleagues’ [4] results show that people tend to more positively rate individuals with a disability in their social acceptability than work performance. In line with these studies, peoples’ awareness about how a colleague with a disability can perform the essential job functions is one potential path to improving their work performance attitudes towards this population as employees. McDonnall et al. [49] demonstrated that a vast majority of people in a work context are not knowledgeable about how people with a disability perform typical job tasks, and this lack of knowledge may negatively affect colleagues’ attitudes. 

Our results also highlighted the effect of educational levels on the attitudes towards people with a disability. This is in line with the study of Le Blanc et al. [50] in which it was emphasized that the greater training of operators leads to more favorable attitudes towards people with disabilities.

Workers that are experienced in working with colleagues with a disability conversely showed a relation with their attitudes. Considerable differences were detected between employees with or with no previous work experience of this type. This result is in line with the study conducted by Hsu et al. [25], which indicated that the participants who had previous contact with workers with a disability tended to have more positive attitudes toward them. One of the primary reasons could have been that this experience may have led them to have more opportunities to interact with people with disabilities in the workplace. This could positively change their attitudes toward workers with a disability due to their acquiring disability awareness through having more opportunities to interact with them [25].

Overall, these results support Stone and Colella’ s model [27], further clarifying the factors affecting the management of individuals with disabilities in a work context. Specifically, according to the model, we highlighted the importance of organizational characteristics and personal features (e.g., gender, nature of the disability, the presentation of disability and previous experience) of the employees with and without disabilities in affecting the attitudes toward work performance and social relations of employees with disabilities.

Practical suggestions. The inclusive work environment should consider the attitudes that colleagues may present towards workers with disabilities that could influence the treatment of people with disabilities at work [51]. Attitudes toward the workers with disabilities may vary with the type of disability, type of presentation of new colleagues with a disability and the attention posed towards their strength, the awareness about work performance social acceptability skills, and previous experience in work with people with a disability. All of these variables should affect and determine the nature of the relationship between colleagues with and without a disability. All of these variables can be addressed by work environment interventions. Therefore, an inclusive work context needs to adapt their strategy to their social inclusion goals [11].

As regards career intervention for work contexts, the organizational inclusion focuses on the elimination of career barriers to empower the full performance to all employees and gives value to the personal differences of employees [52]. It emphasizes that each worker is unique and has the potential to contribute toward the organizations’ purposes [40], so it should be important given support not only to people with a disability and their co-workers but also to all employees with and without a disability [53].

As suggested by Bond and Haynes [54], social workers interested in organizational inclusion should provide multileveled interventions, considering the factors influencing the effective management of diversity and inclusion. A factor refers to the beliefs and stereotypes toward diverse workers, and therefore actions should be undertaken to increase knowledge and awareness about disabilities in general and specifically about employees with a disability who work in the organization. Increased knowledge about their strengths, skills, and values for contributing to professional activities should be a goal. Specifically, information about their work performance and social relationship skills in their previous work experience should be given at the beginning of employment when employees with disabilities are introduced to the work team in order to enhance more positive collegial attitudes [4].

Cramm et al. [54] suggested that job coaching support should be provided not only at the beginning of the work placement and when issues arise, but continuously so as to increase inclusion in the work environment.

Emphasis should be given to the inhibition of social categorization and resultant intergroup biases and the sensitivity to diversity and inclusion, through specific training, team building activities, planning of clear goals about inclusion and their communication in a transparent way at all levels of the organization, and a periodic re-evaluation of policies and strategies of diversity and inclusion goals [54,55].

Managerial strategies and human resource policies that emphasize interdependence and collectivist contexts should also be promoted. Specifically, the values of diversity and collaboration could be encouraged in a wide range of ways, such as by establishing strategies that emphasize the need of a shared mission and a collective value for the contribution of diverse employees, stimulating work teams, communicating clearly that discriminatory behavior is not adequate, and highlighting the benefits of diversity for organizational success [11].

Limitations and future directions. Several limitations of this survey study were identified. First, peoples’ attitudes toward people with disabilities could have been established through cultural beliefs, life experiences, and interactions with them [25]. This means that attitudes toward people with disabilities could change back and forth if people had different contact experiences.

Second, we only engaged employees working in mid-size Italian companies in northeastern Italy. This may influence the generalization of findings to other types of a more structured company. Upcoming researchers who are interested in examining the attitudes of Italian employees toward colleagues with a disability should recruit participants of a more structured company, from other Italian regions, engage other companies and involve participants with different backgrounds, such as consumers and students.

Thirdly, only self-reported data was used to collect data about colleagues’ attitudes. Therefore, in terms of a future direction, researchers could use questionnaires, observations, and face to face interviews as other methods to examine the work and social performance attitudes toward workers with a disability.

## 5. Conclusions

Inclusion concerns the context and the right of all people to participate in an inclusive physical, social and psychological place, guaranteeing every person the active participation in social and civic life. The benefits of work inclusion can be encountered at different levels for individuals with a disability, for the workplace, and the overall social context. If colleagues display inclusive behavior to their colleagues, people with disabilities should become active agents in co-construct their work teams and environments. Therefore, this study was aimed to better understand which variables characterize the employees’ attitudes toward colleagues with adisability based on Stone & Colella’s model and previous studies in this field.

## Figures and Tables

**Table 1:** Means, standard deviations and correlations.

	1	2	3	4	5	6	7
Work performance	1	0.122 **	0.003	0.044	0.075 *	0.066	0.125 **
2. Social relation		1	−0.423 **	0.125 **	−0.004	0.102 **	0.069 *
3. Disability			1	0	0	0	0
4. Gender				1	0.094 **	0.027	0.005
5. Education					1	0.099 **	−0.037
6. Previous experience						1	−0.045
7. Type of presentation							1
*Mean*	20.091	14.39					
*DS*	5.11	3.76					

* *p* < 0.05; ** *p* < 0.01.

**Table 2:** Means and standard deviations in the work performance and social acceptability attitudes with SD, ID, and PP.

	Work Performance		Social Acceptability	
	M	DS	M	DS
Sensory disability	20.08	4.88	15.66	3.01
Intellectual disability	20.08	4.82	15.82	3.24
Psychological problem	20.11	5.30	11.81	3.50

**Table 3:** Regression Models of Type of disability, Gender, Educational Level, Previous Experience and Type of Presentation on Work Performance and Social Acceptability.

	Work Performance		Social Acceptability	
	Model 1		Model 2	
	β	SE	β	SE
Disability	0.001	0.213	−0.423 **	0.143
Gender	0.037	0.352	0.116 **	0.238
Education	0.071 **	0.228	0.025	0.154
Previous experience	0.064	0.368	0.103 **	0.249
Type of presentation	0.128 **	0.347	0.071 **	0.235

** *p* < 0.01.

## Data Availability

Not applicable.
